# Zebra finch song is a very short-range signal in the wild: evidence from an integrated approach

**DOI:** 10.1093/beheco/arab107

**Published:** 2021-09-22

**Authors:** Hugo Loning, Simon C Griffith, Marc Naguib

**Affiliations:** 1 Behavioural Ecology Group, Wageningen University & Research, De Elst 1, 6708 WD Wageningen, The Netherlands; 2 Department of Biological Sciences, Macquarie University, Sydney, NSW 2109, Australia

**Keywords:** active space, animal communication, birdsong, communication distance, social behavior, *Taeniopygia guttata*

## Abstract

Birdsong is typically seen as a long-range signal functioning in mate attraction and territory defense. Among birds, the zebra finch is the prime model organism in bioacoustics, yet almost exclusively studied in the lab. In the wild, however, zebra finch song differs strikingly from songbirds commonly studied in the wild as zebra finch males sing most after mating and in the absence of territoriality. Using data from the wild, we here provide an ecological context for a wealth of laboratory studies. By integrating calibrated sound recordings, sound transmission experiments and social ecology of zebra finches in the wild with insights from hearing physiology we show that wild zebra finch song is a very short-range signal with an audible range of about nine meters and that even the louder distance calls do not carry much farther (up to about fourteen meters). These integrated findings provide an ecological context for the interpretation of laboratory studies of this species and indicate that the vocal communication distance of the main laboratory species for avian acoustics contrasts strikingly with songbirds that use their song as a long-range advertisement signal.

## Teaser

In their natural environment, zebra finches sing softly and their songs do not transmit far. This finding is in sharp contrast with our current understanding of birdsong as a typical form of long-range communication. Since the zebra finch is the main laboratory model for bird acoustics, our findings from zebra finches in the wild combined with data on hearing physiology provide a new ecological perspective on the potential function of zebra finch song.

## Introduction

Animal communication plays an integral role in life history events, such as finding a partner, defending a territory, or warning for predators (Searcy and Nowicki 2005; Bradbury and Vehrenkamp 2011). As a consequence, animals produce a striking diversity in signals, from very subtle short-range signals to conspicuous far-ranging displays like many acoustic and visual advertisement signals, adapted to function in the environment in which they have evolved (Brumm and Naguib 2009). A key factor for a signal to function is that its coded information reaches the intended receiver. Indeed, the active space of a signal (Brenowitz 1982), the distance over which a signal can function, is key in unraveling the function of a signal, as the signal structure and its information at the distance at which a receiver responds pose a primary selection pressure (Gerhardt 1994). Some animals maximize their conspicuousness by using elevated display posts, as used for long distance vocalizations (Sprau et al. 2012), or by seeking display sites that maximize contrast and visibility (Endler and Théry 1996; Uy and Endler 2004). Yet although the active space of a signal is often determined by its amplitude or conspicuousness and the transmission constraints of the physical environment, eventually the sensory and perceptual ability and acuity of the receiver (Wiley and Richards 1978; Naguib and Wiley 2001; Lohr et al. 2003; Gall et al. 2012) need to be considered. This is indeed crucial when assessing which parts of the information emitted by a signaler can be picked up from attenuated and degraded signals after transmission through the environment.

Among animal signals, vocalizations, and specifically birdsong and calls are among the best-studied communication systems, and insights from birdsong have fundamentally shaped the broader view on the evolution of animal communication (Searcy and Nowicki 2005). The most studied functions of birdsong are mate attraction and territory advertisement, yet song can also have more subtle functions affecting daily behavioral routines and decisions among pair members, the wider neighborhood, and socially relevant individuals in groups (Snijders and Naguib 2017). One of the main model bird species is the Australian zebra finch (*Taeniopygia guttata castanotis*), providing the primary avian model organism in laboratory studies world-wide (Griffith and Buchanan 2010; Griffith et al. 2021). Zebra finches have been key in studies on mate choice (Slater et al. 1988; Riebel 2009; Kniel et al. 2015), long term effects of early developmental stress (Spencer et al. 2005; Monaghan et al. 2012; Honarmand et al. 2015), and specifically are a textbook model for the physiology, neurobiology and genetics of the song system (Haesler et al. 2004; Gil et al. 2006; Warren et al. 2010; Ma et al. 2020) including song development and learning (Slater et al. 1988; Kriengwatana et al. 2016; Hauber et al. 2021; Tchernichovski et al. 2021). Yet, very few studies have addressed zebra finch song in the wild (Dunn and Zann 1996a, 1996b; Woodgate et al. 2012), so that the ecological context and perspective on the findings from laboratory studies is largely lacking and often based on anecdotal observations (Immelmann 1968; Zann 1996). Zebra finches are social, non-territorial birds that live in fission-fusion societies in the arid zone of Australia (McCowan et al. 2015; Brandl et al. 2021), and thus are exposed to different selection-pressures, both socially, and environmentally compared with the well-studied temperate forest birds that dominate the literature on bird song (Catchpole and Slater 2008).

Advantages of using zebra finch song under laboratory conditions as a model for animal communication and the neural basis of song learning, are that males sing all year round, individuals sing a unique single motif (song) produced with only small variation across repetitions (Sturdy et al. 1999), and males sing reliably when exposed to females (Riebel 2009). Whilst the latter characteristic has made the zebra finch song a major focus of work on mate choice, wild males continue to sing outside breeding events (Zann 1996; Griffith 2019), and indeed most males are paired for life from an early age (Zann 1996). As such, most of a male’s song is produced after the initial formation of the pair bond. Additionally, extra-pair paternity rates in the wild are low (2.4% in Birkhead et al. 1990; 1.7% in Griffith et al. 2010). These observations question the general assumption that the primary function of song in this species is mate attraction (Griffith 2019). Yet, although in the laboratory birds are usually kept in stable, single sex groups, or pairs, in the wild they live in loose associations, where individuals stay with their partner for life, but with pairs joining and leaving broader social groups on a regular basis (McCowan et al. 2015; Brandl et al. 2021). To understand the function of vocalizations within social groups and their potential role in social facilitation, a key step is to understand the communication range, as it provides the context in which the signal can function within the natural setting and in which it is selected by receiver responses.

Communication range in birds is often studied by combining estimates of sound amplitude of vocalizing individuals with either a modeling approach (Lohr et al. 2003; Nemeth and Brumm 2010; Derryberry et al. 2016, 2020) or sound transmission experiments, in which the sound is broadcast and re-recorded across a range of distances for subsequent acoustic analyses (Brenowitz 1982; Naguib et al. 2008; Gall et al. 2012). Such transmission experiments have been key in discussions on the communication distance, how sounds regulate the spacing of individuals (Wiley and Richards 1978; Waser and Wiley 1979; Bradbury and Vehrenkamp 2011), and on information transfer in social networks among individuals without close spatial associations (Snijders and Naguib 2017). Yet, animals often do not respond to very distant signals, as they appear to be less salient (Naguib and Wiley 2001). Thus, a complementary approach to assess communication distance, next to playback experiments, is to integrate signal broadcast amplitude, with sound transmission experiments and the actual hearing abilities of the receivers. Hearing curves, the sensitivity to different frequencies of a sound, are commonly determined under standardized conditions with psycho-acoustic experiments in the laboratory (Dooling et al. 2000; Henry et al. 2016). Studies in invertebrates in contrast have been able to use the neurobiological responses to sound in the field as a “biological microphone” (Rheinlaender and Römer 1986; Römer 1993, 2021), revealing auditory responses to long distance signals directly under field conditions. Field studies integrating such hearing thresholds in birds have focused mainly on signal detection in noise, an important approach specifically with respect to communication at high environmental or anthropogenic noise levels (Lohr et al. 2003; Nemeth and Brumm 2010; Gall et al. 2012; Derryberry et al. 2016, 2020). Among birds, zebra finches are among the few species in which hearing thresholds as well as masked thresholds, the precise signal-to-noise ratio within relevant frequency bands that still allows for detection, have been determined (Okanoya and Dooling 1987; Prior et al. 2018). Zebra finches thus provide an excellent opportunity to integrate data from hearing thresholds with acoustic signals in the wild, allowing us to fill a major gap in understanding on communication ranges in animals, and in this important model species in particular.

To determine the communication distance of song and distance calls of wild zebra finches we (1) made calibrated recordings to determine natural signaling amplitudes of wild zebra finches, (2) conducted sound transmission experiments of songs and distance calls at their natural amplitude in the native environment in the Australian arid zone and (3) integrated the results with laboratory data on zebra finch hearing physiology. Additionally, we conducted field transects to characterize perch height and the distance between individuals when singing. These integrated approaches provide an important ecological base for understanding the function and evolution of song in the primary laboratory-based avian model organism.

## METHODS

We conducted all fieldwork at Fowlers Gap Arid Zone Research Station, New South Wales, Australia, using a population of nest-box breeding zebra finches (Griffith et al. 2008; Brandl et al. 2019). The areas inhabited by zebra finches typically consist of several creek lines vegetated by widely spaced low bushes such as bluebush (*Maireana* sp.) and low trees and shrubs, such as prickly wattle (*Acacia victoriae*), dead finish (*Acacia tetragonophylla*), boobialla (*Myoporum montanum*) and native apricot (*Pittosporum angustifolium*). There was an ongoing drought during this study and most natural sources of surface water in the surrounding were dry. Water was thus available almost exclusively through livestock troughs.

### Calibrated recordings and amplitude measurements

We recorded wild zebra finch songs and distance calls between 26 September and 31 October 2018 on days with low wind between 08:00 AM and 03:00 PM. Recordings were made opportunistically throughout the study site when the singing individual was in sight, so that we could determine its orientation and distance from the microphone, determined afterwards using a measuring tape. All recordings were made under very low wind conditions and under the extremely low noise levels of the Australian arid zone. For each recorded vocalization we scored the orientation of the bird in relation to the microphone and whether it originated from the focal individual, because recordings were made in social contexts. Zebra finches are mostly seen with their partner or in small groups (McCowan et al. 2015) and their song is not used as an individual territorial advertisement but given in social contexts. All of the opportunistically recorded males were singing with at least one conspecific nearby and we could always clearly identify the singing male due to the short range at which we recorded them. We used directional microphones (Sennheiser MKH60 in MZS 20-1 + MZH + MZW 70-1 basket windscreen and Sennheiser ME66/K6 with foam windscreen) and recorded at 44.1 kHz 16 bit on digital recorders (Tascam DR100-MKIII) with standardized gain (55 dB for both microphones). For each day we recorded a 1 kHz tone (created in Audacity 2.2.2) at 1 m with both microphones (gain also at 55 dB) for sound file calibration. The tone was played at 65 dB (1 m, Voltcraft SL-300 sound pressure level (SPL) meter, A-weighted, slow response, precision ±1.4 dB at 1 kHz) from an Olympus DM-670 recorder through a UE Megaboom loudspeaker, mounted on a tripod at 1.6 m.

In total we recorded 193 distance calls from 40 males and 345 song motifs (Sossinka and Böhner 1980; Sturdy et al. 1999) from 45 males, of which 16 individuals were recorded in a pair context and 33 were recorded in a social context (i.e., more than two other individuals present), with five individuals having been recorded in both contexts and one individual in an undocumented context. To maintain a high degree of accuracy for the amplitude measurements, we used vocalizations only of focal individuals facing the microphone and recorded within eight meters. This resulted in high quality recordings of a total of 26 individuals, of which 10 individuals were recorded in a pair context and 17 in a social context, with one individual having been recorded in both contexts. We measured 5.3 ± 4.0 (mean ± SD, range 1–18, *N* = 26) song motifs per individual, recorded at 3.8 ± 1.2 m (mean ± SD, range 1.8–6.5 m).

All recordings were high-pass filtered (settings: 400 Hz, 48 dB roll-off/octave) in Audacity. Because the relative amplitude of specific elements appears consistent within males (Brumm 2009 and personal observation), we measured the root mean square (RMS) of the loudest 125 ms of a song motif/distance call using the “contrasts” function in Audacity (Brumm 2009 also measured 125 ms). This often corresponded with the duration of the single song element and spanned most of the duration of the distance calls. Per individual male we always selected the same part of the same song element/distance call. The measured values were in decibels relative to full-scale (dBFS), which we then subsequently translated to SPL (all SPL reported are re 20 µPa) using the calibration tones. Other than the high-pass filter, we did not additionally correct for noise, as noise levels at our field sites in the absence of wind were extremely low (see the spectrum-level background noise of our transmission experiment in Results).

Similarly, we A-weight-filtered each calibration tone (“equalization” function in Audacity) and measured the RMS over 125 ms, omitting environmental noise. Because there was variation per day (SD of 2.2 and 1.5 dB for the MKH60 and ME66, respectively) and not all calibration tones were recorded on the same day as the recording days for practical reasons, we averaged these per microphone. This resulted in an overall average calibration value used for all recordings of a specific microphone, the microphone dependent calibration value c_mic_: 65 dB SPL corresponded with −7.6 ± 0.8 dBFS (mean ± SE, *N* = 7) for the MKH60 and with −5.6 ± 0.7 dBFS (mean ± SE, *N* = 5) for the ME66/K6. We obtained the calibrated RMS of the vocalizations in SPL by subtracting this calibration value of the used microphone and adding the sound pressure level of the calibration tone, SPL_tone_, 65 in our case. Then, for each calibrated vocalization RMS value, we calculated the dB level at one meter using spherical spread, 20 * log_10_(d), where d is the recording distance [RMS_calibrated_ = RMS_measured_ – c_mic_ + SPL_tone_ + 20 * log_10_ (d)].

### Transmission experiment and analysis

We conducted transmission experiments on low-wind days between 18 and 30 November 2018, playing seven high-quality recordings of song, male distance calls, and female distance calls each (21 vocalizations in total). Using the RMS in dBFS of the loudest 125 ms (like our amplitude measurements) we normalized all songs to the same amplitude and set both male and female distance calls to be 7.4 dBFS (the amplitude difference between song and distance calls, see Results) louder than the songs. We added a 1 kHz calibration tone that was 14.5 dBFS louder than the songs for calibration purposes.

We broadcast this master file at six locations (with a tripod-mounted UE Megaboom and an Olympus DM-670 recorder, with the speaker center at 1.6 m height, the average perch height of singing individuals in our area, see Results), re-recording it (Sennheiser microphone MKH40 in the basket windscreen; same height as loudspeaker; Tascam DR100-MKIII recorder) at the distance of 1, 2, 4, 8, 16, 32, 64, 128, and 256 meters for each transect. These transects varied in the amount of vegetation to span the range of microhabitats present in the natural environment of this zebra finch population. We broadcast the zebra finch vocalizations at the pre-determined average natural amplitude (see Results) by ensuring a sound pressure level of 65 dB (A-weighted, slow-response) for the 1 kHz calibration tone with our SPL meter. We used a fixed microphone sensitivity (53 dB gain) for all recordings in all transects. Although wind speeds were low during recording days, we still recorded the broadcast master file multiple times at each distance (usually three to four times) to have sufficient repeats for analysis in case of occasional gusts masking a signal.

We manually checked every recorded repetition’s spectrogram to exclude ones with wind or insect noise and cut out each repeat at the same starting point, resulting in aligned sound files with each specific vocalization at a fixed timestamp. Then, we fed these selections through a custom made Matlab script (version 2020b) which applied for every vocalization a series of band-pass filters over the 500–8000 Hz range (in 100 Hz steps), calculating the root-mean-square value (RMS) of the loudest 125 ms for every band. 125 ms corresponds with the “fast” setting of a SPL meter and is a time that falls within the perceptual time integration of zebra finches (Okanoya and Dooling 1990). The band-pass filter was a minimal-order chebyshev1 filter with a passband frequency that corresponded with the critical bandwidth, a stopband frequency of 0.05 times the passband frequency (e.g., from 290 to 300 Hz and from 500 to 510 Hz for a 200 Hz passband), a 0.01 dB passband ripple and a 30 dB stopband attenuation. The critical bandwidth was calculated as 10^(CR/10)^ (Kittel et al. 2002), where CR is the critical ratio in dB calculated as 9.92 * log_10_(frequency) − 4.8 (Okanoya and Dooling 1987). For every band-pass filtered vocalization, we also measured 0.4 seconds of band-passed background noise in the silence after the specific vocalization. Such noise measured over the critical bandwidth functions as the masking threshold (GM Klump, personal communication). This process resulted in a total of *N* = 214 396 spectrum-level amplitude measurements for both vocalizations and background noise (song: *N* = 64 372, male distance calls: *N* = 73 948 and female distance calls: *N* = 76 076).

We calibrated all values to SPL using the 65 dB (A-weighted) reference tone that we recorded. For each transect, we measured the RMS in dBFS of one of these recorded A-weight filtered 1 kHz reference tones at 1 m in Audacity (using the contrasts function and equalization, see above). The resulting calibration value that we added to each amplitude measurement of that transect was therefore: the absolute value of this measured dBFS value of the reference tone +65 (its dB in SPL) –3.01 (the RMS in dBFS of the loudest tone possible).

### Transects

We walked transects in six sites on a weekly basis between 7:30 AM and 5:00 PM from 12 October–4 December in 2018 and 29 August–6 December in 2019. All transects consisted of an observer walking from a local water point used by zebra finches (e.g., a trough, or a water basin) towards a vegetated area with nest boxes, and then continuing in the nestbox area (which often followed creek lines) until the total distance walked was 1 km. Transects followed the same route every time.

When zebra finches were detected, we scored group size and whether there was singing. For singing zebra finches we estimated perch height (2018) or distance to group members (2019). If there were other groups around in other bushes and detected, this was sometimes noted, but not systematically. During 77 of a total 116 transects, we observed zebra finches a total of 265 times, of which 94 observations included singing birds. Of 49 singing birds we scored the perch height of the singing individual to validate our transmission experiment broadcast height. Of 43 singing birds we scored the maximum distance to group members, in other words, the distance between the two birds in a group farthest from each other. This allowed us to estimate zebra finches’ receiver distance in the wild. In seven of these we also estimated the distance of the next nearest group that was detected at the same time. We received approval by the Macquarie University Animal Ethics Committee (Animal Research Authority 2018/027) for all work in this study.

### Statistical analysis

All statistical analyses were conducted in R (version 4.0.2). For calculating the mean vocalization amplitude, we averaged all amplitude values per individual (because of the large variation in number of songs per individual) and then averaged all individuals. To determine inter-individual differences in amplitude we used an ANOVA (function aov) on a dataset which included all vocalizations of the type investigated. To additionally explore whether the two different social contexts (pair or social group) in which we recorded male song, had an effect on song amplitude, we conducted a linear mixed model (function lmer of lme4 package) with song amplitude as a response variable and context (i.e., pair or social) as an explanatory variable and individual as a random effect. To determine the significance of this model, it was compared with the null model (using function anova).

For the transmission experiment, we conducted linear mixed models with the band-passed vocalization amplitude (*N* = 214 396) as a response variable and vocalization-type, doubling of distance, frequency, and background noise amplitude as explanatory variables, with two-way interactions between doubling of distance, frequency, and background noise amplitude, respectively, as well as a three-way interaction between these three factors. Although vocalization-type was our main focus here, we included these other physics-based factors because we can reasonably attribute a large part of the variation in measured amplitude to them, for example (doubling of) distance because sound attenuates over distance; frequency because our broadcasted vocalizations contained relative amplitude differences over the range of frequencies; and background noise because it, even at low amplitudes, should still affect amplitude measurements because sound amplitude is additive (Embleton 1996). The interactions are warranted because frequency-dependent attenuation is expected (frequency * distance), background noise is not flat but biased towards lower frequencies (frequency * noise), and the relative impact of background noise should increase with distance (distance * noise) and this will, too, be frequency-dependent due to the noise bias towards the low end of the spectrum (frequency * distance * noise) (Brumm and Slabbekoorn 2005). We included transect ID (*N* = 6) as a random intercept. This model was the full model, so no stepwise reduction of model parameters was performed (parameter reduction resulted in poorer fits). Subsequently, using the significant model coefficients (±standard errors) of the full model, we modeled the communication distance of the different vocalizations at average natural amplitude with average natural levels of background noise (at -0.93 dB) to calculate at which distance all frequencies of a vocalization were under the absolute hearing threshold of zebra finches (Okanoya and Dooling 1987). We conducted posthoc tests to test for differences in communication distance between the vocalization types (function emmeans of emmeans package).

## RESULTS

### Calibrated recordings

**Figure 1:**
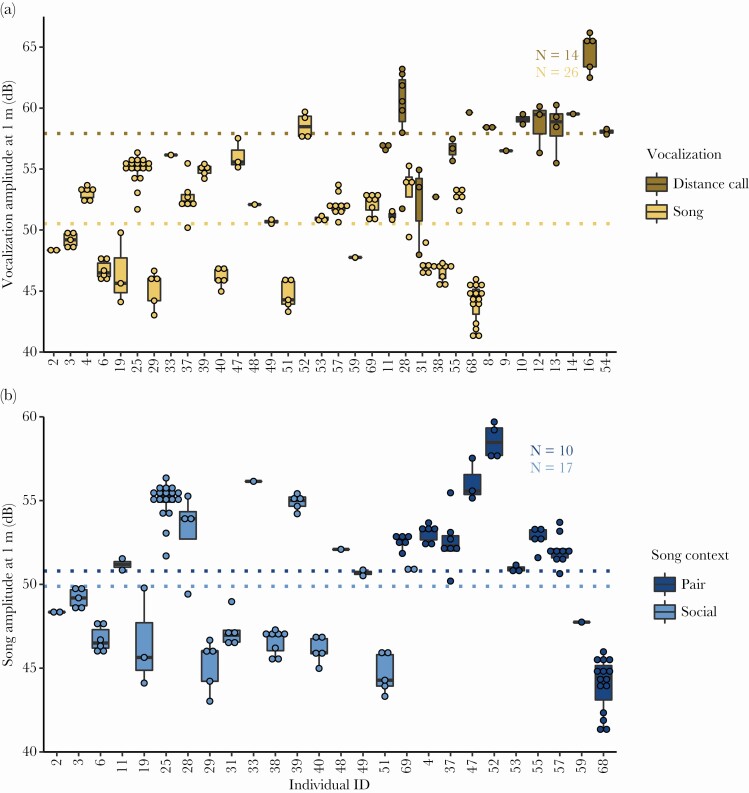
(a) Song and male distance call amplitude (dB re 20 µPa of the loudest 125 ms segment) of calibrated recordings in wild zebra finches. Individuals of which we acquired only song or distance calls are on the left and right side, respectively, with the six individuals of which we acquired both song and distance calls in the middle. (b) Song amplitude (dB re 20 µPa of the loudest 125 ms segment) of the same calibrated recordings in their respective context, which was either pair or social (>2 birds present). For one individual (# 69), we acquired song in both contexts. Points represent songs or distance calls, boxes encompass the first to third quartiles, thick lines are medians and whiskers extend until 1.5 times the inter-quartile range. Dotted lines indicate sample means.

Amplitude at 1 m was 50.5 ± 0.8 dB SPL (mean ± SE, range 44–58.6 dB, *N* = 26, Figure 1a) for songs and 57.9 ± 0.8 dB SPL (mean ± SE, range 52.1–64.6 dB, *N* = 14, Figure 1a) for male distance calls. Individual males varied significantly in their song amplitude (Anova, F_25, 112_ = 66.8, *P* < 0.001, Figure 1a) and distance call amplitude (Anova, F_13, 24_ = 4.5, *P* < 0.001, Figure 1a). The social context also had a small but significant effect on the song amplitude with males in a pair context having sung about 1.9 ± 0.8 dB SPL (mean ± SE) louder than males in a social context (linear mixed model, χ ^2^ = 5.3, *P* = 0.02, pair context *N* = 10, social context *N* = 17, Figure 1b).

### Transmission experiment

**Figure 2:**
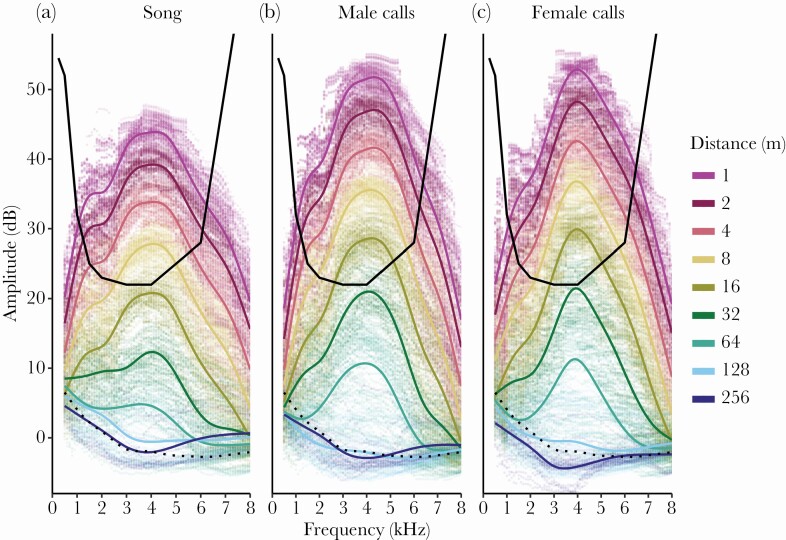
Amplitude (dB re 20 µPa of the loudest 125 ms segment) of wild zebra finch vocalizations (a: song, b: male distance calls, c: female distance calls) transmitted over a 1–256 m distance at average natural amplitude in the natural environment, integrated over critical ratio-based hearing bandwidth. Raw data points (*N* = 214 396) on which the lines are based are shown in corresponding colors. The black line is the audibility curve of (domesticated) zebra finches based on pure tones from Figure 3 in Okanoya and Dooling (1987). The part of the transmitted sound that is above the curve is an approximation for the sound that is audible at that distance by zebra finches. The dotted lines resemble the environmental noise integrated over the respective auditory bandwiths, which is the masking threshold, indicating that masking by environmental noise is not relevant in this environment.

Transmitted zebra finch song, which also was broadcast at lower amplitudes, was significantly softer than male distance calls (Tukey posthoc test, *z*-ratio = 87.635, *N* = 214 396, *P* < 0.001) and male distance calls were softer than female distance calls even though the latter two were broadcast at the same amplitude (Tukey posthoc test, *z*-ratio = 2.881, *N* = 214 396, *P* = 0.011). Considering absolute hearing thresholds of zebra finches (Okanoya and Dooling 1987) and using the significant linear model coefficients (Table 1), all wild zebra finch song produced at average natural amplitude in the natural environment with average spectrum-levels of background noise (−0.93 dB SPL for *N* = 214 396 noise measurements) would not be audible for conspecifics after 8.9 ± 0.7 m (mean ± SE, raw data plotted in Figure 2a). Average male and female distance calls in the same conditions would not be audible after 13.7 ± 1.0 and 13.9 ± 0.9 m, respectively (Figure 2b and c, respectively). The low levels of background noise in the Australian arid zone did not impose limits on zebra finch communication distance (dotted lines in Figure 2).

**Table 1:** Model parameters from the linear mixed model on measured amplitude of the transmitted zebra finch vocalizations in the natural environment, with Transect ID as a random effect (N = 214 396)

Variable	Coefficient	SE	t	P value
Intercept (of female distance call)	47.707	0.460	103.65	<0.001
Doubling of distance [i.e., log_2_(m)]	−6.371	0.018	−347.12	<0.001
Frequency (in kHz)	−1.246	0.015	−84.79	<0.001
Background noise	−1.715	0.010	−164.12	<0.001
Male distance call	−0.121	0.042	−2.88	<0.001
Song	−3.952	0.043	−90.86	<0.001
Doubling of distance * frequency	0.103	0.004	25.38	<0.001
Doubling of distance * noise	0.147	0.004	40.98	<0.001
Frequency * background noise	0.384	0.003	124.22	<0.001
Doubling of distance * frequency * noise	−0.032	0.001	−31.43	<0.001

### Transects

**Figure 3:**
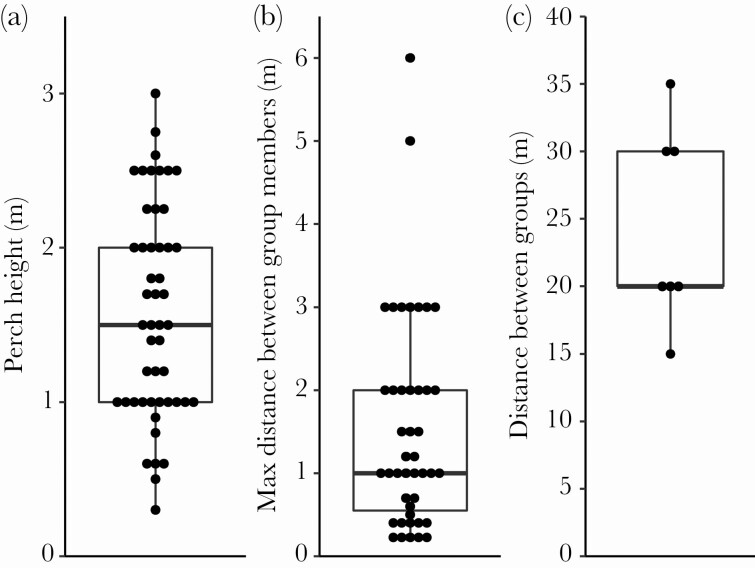
a) Perch height of observed singing zebra finch males during observational transects. b) Maximum distance between group members in zebra finch groups that had one or more singing individuals, a measure of communication distance of wild zebra finch song. c) Distance between zebra finch groups when singing individuals were present, not always scored due to practical constraints. Points represent observations of singing males, boxes encompass the first to third quartiles, thick lines are medians and whiskers extend until 1.5 times the inter-quartile range.

At our study site, which is mostly dominated by low shrubs and trees, the mean perch height of singing birds was 1.6 ± 0.1 m (mean ± SE, range was 0.3–3 m, *N* = 49, Figure 3a). The maximum distance between group members when there was singing (a measure of receiver distance of song) was 1.5 ± 0.2 m (mean ± SE, range was 0.2–6 m, *N* = 43, Figure 3b). The distance of groups with singing zebra finches to closest neighboring groups was 24 ± 3 m (mean ± SE, range was 15–35 m, *N* = 7, Figure 3c). Of 94 song observations, 12 observations (13%) were of males apparently singing alone, 25 observations (27%) were of paired birds and 57 observations (61%) were of groups (range 3–43 individuals, mean ± SD: 12.4 ± 9.0 individuals).

## Discussion

By integrating calibrated sound recordings, sound transmission experiments, and social ecology with insights from hearing physiology we show that wild zebra finch song is a very short-range signal with an estimated audible range of merely 9 m and that even the louder distance calls do not carry much farther (up to about 14 m). These findings are in line with the results of our transects showing that singing individuals are in more than 85% of the observations in very close proximity to conspecifics and they demonstrate that vocalizations would not be heard by birds gathering in the wider vicinity. Together these results shed new light on the communication distance and thus the potential function of vocalizations in one of the main study species for avian acoustics around the world (Griffith and Buchanan 2010; Hauber et al. 2021). By relating our findings with well-established studies on hearing physiology of this prime model organism on avian acoustics in the lab, we are able in an unprecedented way to make much more accurate estimates on vocal communication distance in animals, and specifically with this integration obtain a better understanding of the ecology of avian acoustics.

These integrated findings caution general interpretations about animal communication range as the perceptual system is often not fully integrated in studies on communication distance or assumed to be similar to the human perceptual system, as discussed by Caves et al. (2019). Studies that did consider the hearing mainly focused on the effects of noise on signal detection (Nemeth and Brumm 2010; Gall et al. 2012; Derryberry et al. 2016, 2020). Our findings that singing zebra finches can be heard by other conspecifics over merely a few meters at extremely low noise levels show that the song of this model species is very different in communication range, and thus potential function compared with the widely studied territorial song of temperate zone songbirds. Classically, birdsong is seen as long-distance advertisement signal (Brumm and Naguib 2009), and sound transmission experiments have been used to determine the space across which the signal can function (Richards 1981; Dabelsteen et al. 1993), leading to transmission ranges of 100 and more meters (Naguib et al. 2008). Even in our own sound transmission experiment shown here, we reveal that the rather soft song and the slightly louder distance calls can be re-recorded over a substantial range (64+ m), albeit in a low-noise environment (dotted line Figure 2). Yet, combining these physical measurements with the relevant hearing curve and critical ratio function (the signal-to-noise ratio at masking threshold within a particular frequency band), derived from a controlled laboratory experiment (Okanoya and Dooling 1987), shows that sound transmission experiments alone can be very misleading by overestimating the communication range. This overestimation of communication distance is striking when comparing our calculated ±14 m distance call detection threshold with the previous experiment by Mouterde et al. (2014), where zebra finch calls could still be discriminated at 256 m distance by applying sophisticated software (or 64 m when corrected for the 12 dB higher broadcast amplitude in their study), but zebra finch auditory capabilities were not considered.

Despite having a very high temporal hearing resolution (Dooling and Lohr 2006; Lohr et al. 2006; Prior et al. 2018), zebra finches, like many other birds, have a higher overall hearing threshold and narrower audible frequency range compared with humans (Dooling and Saunders 1975; Dooling 1982, 1992), thus are less sensitive in detecting sound than humans are. This knowledge on hearing thresholds is not new (Dooling 1982), yet has rarely been integrated with ecological field acoustics (but see Klump et al. 1986; Henry and Lucas 2008; Gall et al. 2012; Derryberry et al. 2016; Henry et al. 2016). By integrating hearing thresholds our data shed a different light on conclusions drawn from previous sound transmission experiments which determined very long communication ranges in animal vocalizations (Naguib et al. 2008; Mouterde et al. 2014). Because, in our study the hearing curve was taken from laboratory experiments with a different population and sounds, we need to consider that some birds hear better than others and that hearing curves of animals in the wild might vary more (Henry et al. 2016). However, due to the very low vocalization amplitudes of wild individuals we found, hearing thresholds would need to be drastically lower to qualify as long-range communication in their spacious native environment. Even if hearing thresholds would be a 10 dB lower in wild bird than in domesticated birds, the audible range for song would still be very short at 28 m (calculated from model parameters in Table 1). To further specify the communication range, ideally also the hearing curve of wild zebra finches should be measured. Moreover, testing the distance at which receivers respond to playback experiments in the field would be very interesting for future studies. Yet, because zebra finches are not territorial, typical strong responses to playback are not expected. A lack of response, however, then cannot simply be attributed to the communication range as an individual that detected the playback, may decide not to respond due to the perceived distance (Naguib and Wiley 2001). Therefore, such playback experiments would require either sophisticated sensors, such as heart-rate monitors, or very specific contexts, such as during mate separation, when females may be specifically responsive when searching their partner.

Our field observations are in line with the calculated very short communication range as zebra finches mostly sang when in pairs or groups, usually within 1.5 m of each other, much closer than the 9 m we calculated. Indeed, our hearing range estimate is conservative because we measured the loudest sections of the song, reflecting primarily the detection range. Zebra finch song is dynamic, with low-amplitude elements being much softer than their loudest elements (Brumm 2009; Ritschard and Brumm 2011). Individual recognition and extraction of subtle information coded in repertoire size or element structures (Woodgate et al. 2012) would require a receiver to be much closer (Lohr et al. 2003; Wiley 2006), and with this knowledge, it may be not so surprising that, despite being unpaired, most males would not sing for females at distances of 3 m in the study by Brumm and Slater (2006). Such variation in the transmission range of different signal components has been shown previously in nightingales (*Luscinia megarhynchos*) in which the bandwidth of broadband trills does not even transmit to the nearest neighbor, whereas whistle-like structures transmit across multiple territories (Naguib et al. 2008). Furthermore, given that hearing thresholds for higher frequencies generally are higher, the difference in the audible range of high frequency components and medium-frequency sounds which mainly fall in the typical range of highest avian hearing sensitivity of 2–4 kHz (Dooling et al. 2000), would even be larger. Because information about condition and arousal can be coded in such subtle features (Perez et al. 2012), such specifics about an individual’s state are likely unavailable at the, often larger, communication distances in widely spaced animals.

With an average difference of 14.5 dB between our calibrated recordings and those of domesticated zebra finches by Brumm (2009), our field recordings revealed much lower amplitudes of the loudest song elements (at 50.5 dB) compared with his recordings in the lab (Brumm 2009: Figure 5, with an average of 71 dB, where amplitude was measured at 50 cm, thus would be 6 dB lower at 1 m). Likewise, the 50.5 dB song amplitude we measured is much lower than the 74–100 dB range of 17 previously reported territorial songbird species (Brackenbury 1979). Although song amplitude itself seems to be affected by vocal learning, but is not particularly heritable in zebra finches (Ritschard and Brumm 2011), there could be several reasons for the strikingly lower amplitudes measured in the wild compared with the lab. First of all, domesticated birds typically are heavier and larger than wild-type birds (Sossinka 1982; Forstmeier et al. 2007). Although Brumm (2009) did not detect a relation between body size and song amplitude within domesticated zebra finches, differences between wild and domesticated zebra finches might be more pronounced despite the large individual variation in song amplitude present in both captive (Brumm and Slater 2006; Brumm 2009) and wild birds (Figure 1a). Such potential song amplitude differences between wild and domesticated birds remain to be tested. Secondly, the singing males in the study by Brumm (2009) were unpaired and housed in single-sex groups before being exposed to a female for song recording. Thus they likely were extremely motivated to sing for mate attraction, whereas most singing adult birds in the wild are likely to be paired, as that is the normal state for a wild adult zebra finch (McCowan et al. 2015). Moreover, next to potential differences in wild and domesticated birds, we cannot discount that the wild birds in our study potentially were in poorer condition, and due to the long-term drought were not breeding during the period of data collection, when they would in better years. Therefore, we cannot rule out that the wild birds might have sung at lower amplitudes than wild birds in better conditions would, although condition alone cannot explain the 14.5 dB difference between our study and (Brumm 2009), since Ritschard and Brumm (2012) observed an amplitude difference of about 4 dB between diet-restricted and control birds. Similarly to Brumm and Slater (2006) and Brumm (2009), we found high variation in song amplitude across individuals, which we expect to be an important driver of variation in communication distance in this species, potentially signaling condition (Ritschard et al. 2010), although this remains to be tested in the wild. Yet, combined with the hearing curves, louder than average singing zebra finches would still not be heard substantially farther, presumably only on few occasions reaching individuals outside their current social group. Specially so in the wide open Australian arid zone with widely spaced vegetation where zebra finches are usually very close to each other or very far apart and outside of hearing range as our transect observations show.

Finally, singing at low amplitude can be considered as an adaptation to the short communication distance when receivers are nearby, as it is the case for the highly social zebra finch. Low amplitude song also occurs in other bird species, where it is usually termed soft song (Reichard and Anderson 2015). Such soft song is a context-dependent low amplitude signal that has been seen as an adaptation to prevent eavesdropping from distant individuals when the signal is intended only for a receiver nearby (Dabelsteen et al. 1998; Rice et al. 2013; Zollinger and Brumm 2015; Ali and Anderson 2018). Such soft song is indeed common in territorial bird species with otherwise loud territorial advertisement song (Anderson et al. 2008; Reichard and Anderson 2015). Blackbirds (*Turdus merula*) for instance produce soft song during times of high arousal during territorial intrusions, referred to as strangled song, possibly to actively limit the signaling range (Dabelsteen and Pedersen 1990). Likewise, dark eyed juncos *(Junco hyemalis*) respond more strongly to soft song than to louder song (Reichard et al. 2011). Yet, as in blackbirds, the soft song in dark-eyed juncos also differs in structure from the louder song so is more than just a soft version of an otherwise louder song. Indeed, Reichard et al. (2011) showed in dark-eyed juncos that the differential response is more linked to the structure of the soft song than to its amplitude. Finally, in these, and other species in which soft song has been observed, the soft song is easily overlooked and typically not the most frequent song that would be opportunistically encountered (Reichard and Anderson 2015). These examples of soft song in other species therefore represent something quite different from the low amplitude singing we have characterized in zebra finches. Zebra finches, despite the variation in singing amplitude within and between individuals, do not produce structurally different songs in different contexts (despite adding extra initial elements on some occasions; Sossinka and Böhner 1980; Sturdy et al. 1999). We did find that males sang slightly louder in pairs than when in social groups, suggesting some degree of context-dependent adjustment of signaling amplitude as shown in domesticated zebra finches (Cynx and Gell 2004; Brumm and Slater 2006). However, our average 1.9 dB context-related difference contrasts with typical differences between quiet and broadcast song. For instance, in song sparrows (*Melospiza melodia*), soft song ranged from 55 to 77 dB whereas “regular” song ranged from 78 to 85 dB (Anderson et al. 2008). The low amplitude of zebra finch song in both contexts thus appears functionally distinct from the context-dependent soft song of territorial bird species. Of course, we cannot completely rule out that zebra finches in some contexts could potentially utter much louder songs or calls, but there is currently no evidence for this in either our data or, as far as we know, in the extensive song literature. This is relevant when using the findings from zebra finch song from lab studies to generalize to other songbirds, as the overall communication range along with the social and functional context in which the song system has evolved, is different from most other species used for avian song research in the wild.

Taken together, the integrated findings imply that song is a within-group signal and information transmitted by singing males can be used only after birds have gathered at close range, and thus cannot drive spatial movements at a larger scale as shown for the loud territorial song of other songbirds (Snijders and Naguib 2017; Bircher et al. 2020). Early in life when pairs form (Zann 1996), a singing male will only reach a female which is already present in the same group, not attract a mate from the distance, as in other songbirds (Catchpole and Slater 2008). Therefore, the song is best considered as part of a sexual display to the multiple individuals that are already in close proximity, and many of which will also be singing, making the mate choice context much more complex than in species in which the signal is used to attract potential mates to a unique location from the distance. Although such vocalizations and the complex multimodal displays in groups have been evident in animals, and specifically so in aviary kept zebra finches for a long time (Immelmann 1968), the ecological implications highlighted here are quite fundamental. In the open landscape of the Australian arid zone, zebra finches split up and reunite frequently (McCowan et al. 2015). Although acoustic signals can be key to guide the spatial movements of animals (Waser and Wiley 1979; Whitehead 1987; Wilczynski and Brenowitz 1988), this is apparently limited to a small spatial scale in zebra finches. Even their relatively soft distance calls are not suited to attract others over long distances. Because vocal signals are of limited use for finding a lost partner or other group members in their vast habitat and home range, they must have evolved other mechanisms underlying their dynamic social organization. The use of stable water sources, specific habitat features, or regular flight routes along creeks as well as joint breeding are such potential adaptations that can facilitate joining others to form temporary groups for roosting or foraging.

In summary, integrating knowledge on the perceptual and processing mechanisms in a broader sense will substantially enhance our understanding about the ecological conditions in which signals have evolved. Likewise, understanding the ecological conditions in which signals function, provides a relevant framework for interpreting mechanistic studies conducted under controlled laboratory conditions.

## Data Availability

Analyses reported in this article can be reproduced using the data provided by Loning et al. (2021).

## References

[CIT0001] Ali S , Anderson R. 2018. Song and aggressive signaling in Bachman’s Sparrow. Auk. 135(3):521–533.

[CIT0002] Anderson RC , Searcy WA, Peters S, Nowicki S. 2008. Soft song in song sparrows: acoustic structure and implications for signal function. Ethology. 114(7):662–676.

[CIT0003] Bircher N , van Oers K, Hinde CA, Naguib M. 2020. Extraterritorial forays by great tits are associated with dawn song in unexpected ways. Behav Ecol. 31:873–883. 32760175 10.1093/beheco/araa040 PMC7390995

[CIT0004] Birkhead TR , Burke T, Zann RA, Hunter FM, Krupa AP. 1990. Extra-pair paternity and intraspecific brood parasitism in wild zebra finches Taeniopygia guttata, revealed by DNA fingerprinting. Behav Ecol Sociobiol. 27(5):315–324.

[CIT0005] Brackenbury J . 1979. Power capabilities of the avian sound-producing system. J Exp Biol. 78:163–166.

[CIT0006] Bradbury JW , Vehrenkamp SL. 2011. Principles of animal communication. Sunderland, MA: Sinauer Associates.

[CIT0007] Brandl HB , Griffith SC, Farine DR, Schuett W. 2021. Wild zebra finches that nest synchronously have long-term stable social ties. J Anim Ecol. 90:76–86. 31407336 10.1111/1365-2656.13082

[CIT0008] Brandl HB , Griffith SC, Schuett W. 2019. Wild zebra finches choose neighbours for synchronized breeding. Anim Behav. 151:21–28.

[CIT0009] Brenowitz EA . 1982. The active space of red-winged blackbird song. J Comp Physiol A. 147(4):511–522.

[CIT0010] Brumm H . 2009. Song amplitude and body size in birds. Behav Ecol Sociobiol. 63(8):1157–1165. 10.1007/s00265-009-0749-y PMC2699386 19554102

[CIT0011] Brumm H , Naguib M. 2009. Environmental acoustics and the evolution of bird song. Adv Study Behav. 40:1–33.

[CIT0012] Brumm H , Slabbekoorn H. 2005. Acoustic communication in noise. Adv Study Behav. 35(05):151–209.

[CIT0013] Brumm H , Slater PJB. 2006. Animals can vary signal amplitude with receiver distance: evidence from zebra finch song. Anim Behav. 72(3):699–705.

[CIT0014] Catchpole CK , Slater PJB. 2008. Bird song: biological themes and variations. Cambridge: Cambridge University Press.

[CIT0015] Caves EM , Nowicki S, Johnsen S. 2019. Von Uexküll revisited: addressing human biases in the study of animal perception. Integr Comp Biol. 59:1451–1462. 31127268 10.1093/icb/icz073

[CIT0016] Cynx J , Gell C. 2004. Social mediation of vocal amplitude in a songbird, *Taeniopygia guttata*. Anim Behav. 67(3):451–455.

[CIT0017] Dabelsteen T , Larsen ON, Pedersen SB. 1993. Habitat-induced degradation of sound signals: quantifying the effects of communication sounds and bird location on blur ratio, excess attenuation, and signal-to-noise ratio in blackbird song. J Acoust Soc Am. 93(4):2206–2220.

[CIT0018] Dabelsteen T , McGregor PK, Lampe HM, Langmore NE, Holland J. 1998. Quiet song in song birds: an overlooked phenomenon. Bioacoustics. 9(2):89–105.

[CIT0019] Dabelsteen T , Pedersen SB. 1990. Song and information about aggressive responses of blackbirds, *Turdus merula*: evidence from interactive playback experiments with territory owners. Anim Behav. 40:1158–1168.

[CIT0020] Derryberry EP , Danner RM, Danner JE, Derryberry GE, Phillips JN, Lipshutz SE, Gentry K, Luther DA. 2016. Patterns of song across natural and anthropogenic soundscapes suggest that white-crowned sparrows minimize acoustic masking and maximize signal content. PLoS One. 11:e0154456. 27128443 10.1371/journal.pone.0154456 PMC4851413

[CIT0021] Derryberry EP , Phillips JN, Derryberry GE, Blum MJ, Luther D. 2020. Singing in a silent spring: birds respond to a half-century soundscape reversion during the COVID-19 shutdown. Science. 370:575–579. 32972991 10.1126/science.abd5777

[CIT0022] Dooling RJ . 1982. Auditory perception in birds. In: Kroodsma DE, Miller E, Ouellet H, editors. Acoustic communication in birds. New York, NY: Academic Press. p. 95–130.

[CIT0023] Dooling RJ . 1992. Hearing in birds. In: Webster DB, Fay RR, Popper AN, editors. The evolutionary biology of hearing. New York, NY: Springer. p. 545–559.

[CIT0024] Dooling RJ , Lohr B, Dent ML. 2000. Hearing in birds and reptiles. In: Dooling RJ, Fay RR, Popper AN, editors. Comparative hearing: birds and reptiles. New York, NY: Springer. p. 308–359.

[CIT0025] Dooling RJ , Lohr B. 2006. Auditory temporal resolution in the zebra finch *(Taeniopygia guttata)*: a model of enhanced temporal acuity. Ornithol Sci. 5(1):15–22.

[CIT0026] Dooling RJ , Saunders JC. 1975. Hearing in the parakeet (*Melopsittacus undulatus*): absolute thresholds, critical ratios, frequency difference limens, and vocalizations. J Comp Physiol Psychol. 88:1–20. 1120787 10.1037/h0076226

[CIT0027] Dunn AM , Zann RA. 1996a. Undirected song encourages the breeding female Zebra finch to remain in the nest. Ethology. 102(4):540–548.

[CIT0028] Dunn AM , Zann RA. 1996b. Undirected song in wild zebra finch flocks: contexts and effects of mate removal. Ethology. 102(4):529–539.

[CIT0029] Embleton TFW . 1996. Tutorial on sound propagation outdoors. J Acoust Soc Am. 100(1):31.

[CIT0030] Endler JA , Théry M. 1996. Interacting effects of lek placement, display behavior, ambient light, and color patterns in three neotropical forest-dwelling birds. Am Nat. 148(3):421–452.

[CIT0031] Forstmeier W , Segelbacher G, Mueller JC, Kempenaers B. 2007. Genetic variation and differentiation in captive and wild zebra finches (*Taeniopygia guttata*). Mol Ecol. 16:4039–4050. 17894758 10.1111/j.1365-294X.2007.03444.x

[CIT0032] Gall MD , Ronald KL, Bestrom ES, Lucas JR. 2012. Effects of habitat and urbanization on the active space of brown-headed cowbird song. J Acoust Soc Am. 132:4053–4062. 23231134 10.1121/1.4764512

[CIT0033] Gerhardt HC . 1994. The evolution of vocalization in frogs and toads. Annu Rev Ecol Syst. 25(1):293–324.

[CIT0034] Gil D , Naguib M, Riebel K, Rutstein A, Gahr M. 2006. Early condition, song learning, and the volume of song brain nuclei in the zebra finch (*Taeniopygia guttata*). J Neurobiol. 66:1602–1612. 17058194 10.1002/neu.20312

[CIT0035] Griffith SC . 2019. Cooperation and coordination in socially monogamous birds: moving away from a focus on sexual conflict. Front Ecol Evol. 7:455.

[CIT0036] Griffith SC , Buchanan KL. 2010. The zebra finch: the ultimate Australian supermodel. Emu. 110(3):v–xii.

[CIT0037] Griffith SC , Holleley CE, Mariette MM, Pryke SR, Svedin N. 2010. Low level of extrapair parentage in wild zebra finches. Anim Behav. 79(2):261–264.

[CIT0038] Griffith SC , Pryke SR, Mariette MM. 2008. Use of nest-boxes by the zebra finch *(Taeniopygia guttata)*: implications for reproductive success and research. Emu. 108(4):311–319.

[CIT0039] Griffith SC , Ton R, Hurley LL, McDiarmid CS, Pacheco-Fuentes H. 2021. The ecology of the zebra finch makes it a great laboratory model but an outlier amongst passerine birds. Birds. 2(1):60–76.

[CIT0040] Haesler S , Wada K, Nshdejan A, Morrisey EE, Lints T, Jarvis ED, Scharff C. 2004. FoxP2 expression in avian vocal learners and non-learners. J Neurosci. 24:3164–3175. 15056696 10.1523/JNEUROSCI.4369-03.2004 PMC6730012

[CIT0041] Hauber ME , Louder MI, Griffith SC. 2021. The natural history of model organisms: neurogenomic insights into the behavioral and vocal development of the zebra finch. Elife. 10:e61849. 34106827 10.7554/eLife.61849 PMC8238503

[CIT0042] Henry KS , Gall MD, Vélez A, Lucas JR. 2016. Avian auditory processing at four different scales: variation among species, seasons, sexes, and individuals. In: Bee MA, Miller CT, editors. Physiological mechanisms in animal communication. Cham, Zwitserland: Springer International Publishing. p. 17–54.

[CIT0043] Henry KS , Lucas JR. 2008. Coevolution of auditory sensitivity and temporal resolution with acoustic signal space in three songbirds. Anim Behav. 76(5):1659–1671.

[CIT0044] Honarmand M , Riebel K, Naguib M. 2015. Nutrition and peer group composition in early adolescence: Impacts on male song and female preference in zebra finches. Anim Behav. 107:147–158.

[CIT0045] Immelmann K . 1968. Zur biologischen Bedeutung des Estrildidengesanges. J Ornithol. 109(3):284–299.

[CIT0046] Kittel M , Wagner E, Klump GM. 2002. An estimate of the auditory-filter bandwidth in the Mongolian gerbil. Hear Res. 164:69–76. 11950526 10.1016/s0378-5955(01)00411-7

[CIT0047] Klump GM , Kretzschmar E, Curio E. 1986. The hearing of an avian predator and its avian prey. Behav Ecol Sociobiol. 18(5):317–323.

[CIT0048] Kniel N , Dürler C, Hecht I, Heinbach V, Zimmermann L, Witte K. 2015. Novel mate preference through mate-choice copying in zebra finches: sexes differ. Behav Ecol. 26(2):647–655.

[CIT0049] Kriengwatana B , Spierings MJ, ten Cate C. 2016. Auditory discrimination learning in zebra finches: effects of sex, early life conditions and stimulus characteristics. Anim Behav. 116:99–112.

[CIT0050] Lohr B , Dooling RJ, Bartone S. 2006. The discrimination of temporal fine structure in call-like harmonic sounds by birds. J Comp Psychol. 120:239–251. 16893261 10.1037/0735-7036.120.3.239

[CIT0051] Lohr B , Wright TF, Dooling RJ. 2003. Detection and discrimination of natural calls in masking noise by birds: estimating the active space of a signal. Anim Behav. 65(4):763–777.

[CIT0052] Loning H , Griffith SC, Naguib M. 2021. Data from: zebra finch song is a very short-range signal in the wild: evidence from an integrated approach. Behav Ecol. doi:10.5061/dryad.xgxd254h9. 10.1093/beheco/arab107 PMC8857932 35197805

[CIT0053] Ma S , Ter Maat A, Gahr M. 2020. Neurotelemetry reveals putative predictive activity in HVC during call-based vocal communications in Zebra finches. J Neurosci. 40:6219–6227. 32661023 10.1523/JNEUROSCI.2664-19.2020 PMC7406282

[CIT0054] McCowan LSC , Mariette MM, Griffith SC. 2015. The size and composition of social groups in the wild zebra finch. Emu. 115(3):191–198.

[CIT0055] Monaghan P , Heidinger BJ, D’Alba L, Evans NP, Spencer KA. 2012. For better or worse: reduced adult lifespan following early-life stress is transmitted to breeding partners. Proc R Soc B Biol Sci. 279(1729):709–714. 10.1098/rspb.2011.1291 PMC3248736 21849320

[CIT0056] Mouterde SC , Theunissen FE, Elie JE, Vignal C, Mathevon N. 2014. Acoustic communication and sound degradation: how do the individual signatures of male and female zebra finch calls transmit over distance? PLoS One. 9:e102842. 25061795 10.1371/journal.pone.0102842 PMC4111290

[CIT0057] Naguib M , Schmidt R, Sprau P, Roth T, Flörcke C, Amrhein V. 2008. The ecology of vocal signaling: male spacing and communication distance of different song traits in nightingales. Behav Ecol. 19(5):1034–1040.

[CIT0058] Naguib M , Wiley RH. 2001. Review: estimating the distance to a source of sound: mechanisms and adaptations for long-range communication. Anim Behav. 62(5):825–837.

[CIT0059] Nemeth E , Brumm H. 2010. Birds and anthropogenic noise: are urban songs adaptive? Am Nat. 176:465–475. 20712517 10.1086/656275

[CIT0060] Okanoya K , Dooling RJ. 1987. Hearing in passerine and psittacine birds: a comparative study of absolute and masked auditory thresholds. J Comp Psychol. 101:7–15. 3568610

[CIT0061] Okanoya K , Dooling RJ. 1990. Temporal integration in zebra finches (*Poephila guttata*). J Acoust Soc Am. 87:2782–2784. 2373807 10.1121/1.399069

[CIT0062] Perez EC , Elie JE, Soulage CO, Soula HA, Mathevon N, Vignal C. 2012. The acoustic expression of stress in a songbird: does corticosterone drive isolation-induced modifications of zebra finch calls? Horm Behav. 61:573–581. 22387308 10.1016/j.yhbeh.2012.02.004

[CIT0063] Prior NH , Smith E, Lawson S, Ball GF, Dooling RJ. 2018. Acoustic fine structure may encode biologically relevant information for zebra finches. Sci Rep. 8:6212. 29670131 10.1038/s41598-018-24307-0 PMC5906677

[CIT0064] Reichard DG , Anderson RC. 2015. Why signal softly? The structure, function and evolutionary significance of low-amplitude signals. Anim Behav. 105:253–265.

[CIT0065] Reichard DG , Rice RJ, Vanderbilt CC, Ketterson ED. 2011. Deciphering information encoded in birdsong: male songbirds with fertile mates respond most strongly to complex, low-amplitude songs used in courtship. Am Nat. 178:478–487. 21956026 10.1086/661901

[CIT0066] Rheinlaender J , Römer H. 1986. Insect hearing in the field I. The use of identified nerve cells as “biological microphones”. J Comp Physiol A. 158:647–651.

[CIT0067] Rice RJ , Schrock SE, Reichard DG, Schultz EM. 2013. Low-amplitude songs produced by male dark-eyed juncos (*Junco hyemalis*) differ when sung during intra- and inter-sexual interactions. Behaviour. 150:1183–1202.

[CIT0068] Richards DG . 1981. Estimation of distance of singing conspecifics by the Carolina wren. Auk. 98(1):127–133.

[CIT0069] Riebel K . 2009. Song and female mate choice in zebra finches: a review. Adv Study Behav. 40:197–238.

[CIT0070] Ritschard M , Brumm H. 2011. Effects of vocal learning, phonetics and inheritance on song amplitude in zebra finches. Anim Behav. 82(6):1415–1422.

[CIT0071] Ritschard M , Brumm H. 2012. Zebra finch song reflects current food availability. Evol Ecol. 26(4):801–812.

[CIT0072] Ritschard M , Riebel K, Brumm H. 2010. Female zebra finches prefer high-amplitude song. Anim Behav. 79(4):877–883.

[CIT0073] Römer H . 1993. Environmental and biological constraints for the evolution of long- range signalling and hearing in acoustic insects. Philos Trans R Soc Lond B. 340(1292):179–185.

[CIT0074] Römer H . 2021. Neurophysiology goes wild: from exploring sensory coding in sound proof rooms to natural environments. J Comp Physiol A Neuroethol Sens Neural Behav Physiol. 207:303–319. 33835199 10.1007/s00359-021-01482-6 PMC8079291

[CIT0075] Searcy WA , Nowicki S. 2005. The evolution of animal communication: reliability and deception in signaling systems. Princeton, NJ: Princeton University Press.

[CIT0076] Slater PJB , Eales LA, Clayton NS. 1988. Song learning in zebra finches *(Taeniopygia guttata):* progress and prospects. Adv Study Behav. 18:1–34.

[CIT0077] Snijders L , Naguib M. 2017. Communication in animal social networks. Adv Study Behav. 49:297–359.

[CIT0078] Sossinka R . 1982. Domestication in birds. In: Farner D, King J, Parkes K, editors. Avian biology. New York, NY: Academic Press. p. 373–403.

[CIT0079] Sossinka R , Böhner J. 1980. Song types in the zebra finch *Poephila guttata castanotis*. Z Tierpsychol. 53(2):123–132.

[CIT0080] Spencer KA , Wimpenny JH, Buchanan KL, Lovell PG, Goldsmith AR, Catchpole CK. 2005. Developmental stress affects the attractiveness of male song and female choice in the zebra finch *(Taeniopygia guttata)*. Behav Ecol Sociobiol. 58(4):423–428.

[CIT0081] Sprau P , Roth T, Naguib M, Amrhein V. 2012. Communication in the third dimension: song perch height of rivals affects singing response in nightingales. PLoS One. 7:e32194. 22448215 10.1371/journal.pone.0032194 PMC3308953

[CIT0082] Sturdy CB , Phillmore LS, Weisman RG. 1999. Note types, harmonic structure, and note order in the songs of zebra finches. J Comp Psychol. 113(2):194–203.

[CIT0083] Tchernichovski O , Eisenberg-Edidin S, Jarvis ED. 2021. Balanced imitation sustains song culture in zebra finches. Nat Commun. 12(1):1–14. 33963187 10.1038/s41467-021-22852-3 PMC8105409

[CIT0084] Uy JAC , Endler JA. 2004. Modification of the visual background increases the conspicuousness of golden-collared manakin displays. Behav Ecol. 15(6):1003–1010.

[CIT0085] Warren WC , Clayton DF, Ellegren H, Arnold AP, Hillier LW, Künstner A, Searle S, White S, Vilella AJ, Fairley S, et al. 2010. The genome of a songbird. Nature. 464:757–762. 20360741 10.1038/nature08819 PMC3187626

[CIT0086] Waser PM , Wiley RH. 1979. Mechanisms and evolution of spacing in animals. In: Marler P, Vandenbergh J, editors. Social behavior and communication. Boston, MA: Springer. p. 159–223.

[CIT0087] Whitehead JM . 1987. Vocally mediated reciprocity between neighbouring groups of mantled howling monkeys, *Alouatta palliata palliata*. Anim Behav. 35(6):1615–1627.

[CIT0088] Wilczynski W , Brenowitz EA. 1988. Acoustic cues mediate inter-male spacing in a neotropical frog. Anim Behav. 36(4):1054–1063.

[CIT0089] Wiley RH . 2006. Signal detection and animal communication. Adv Study Behav. 36:217–247.

[CIT0090] Wiley RH , Richards DG. 1978. Physical constraints on acoustical communication in the atmosphere: implications for the evolution of animal vocalizations. Behav Ecol Sociobiol. 3:69–94.

[CIT0091] Woodgate JL , Mariette MM, Bennett ATD, Griffith SC, Buchanan KL. 2012. Male song structure predicts reproductive success in a wild zebra finch population. Anim Behav. 83(3):773–781.

[CIT0092] Zann RA . 1996. The zebra finch: a synthesis of field and laboratory studies. New York, NY: Oxford University Press.

[CIT0093] Zollinger SA , Brumm H. 2015. Why birds sing loud songs and why they sometimes don’t. Anim Behav. 105:289–295.

